# Psychosocial and Mental Health Indicators and Viral Non-Suppression Among Adults Receiving Antiretroviral Therapy in Gaza Province, Mozambique: A Cross-Sectional Study

**DOI:** 10.3390/healthcare14213679

**Published:** 2026-10-30

**Authors:** Ana Teresa Quissico, Isabelle Munyangaju, Vanilo Nhone, Ismail Chiposse, Sérgio Renaldo João, Izaidino Muchanga, Mulássua Simango, Marcelina Lindonde, Sílvio Alexandre, Amâncio Nhangave

**Affiliations:** 1 District Health Service, Women and Social Action, Xai-Xai P.O. Box 33, Mozambique; anateresaraimundo2001@gmail.com (A.T.Q.); www.nhonej21@gmail.com (V.N.); 2 Tinpswalo Research Association to Fight AIDS and TB, Chokwe P.O. Box 35, Mozambique; 3 Núcleo de Pesquisa Pediatria, Faculdade de Medicina, Universidade Eduardo Mondlane, Maputo P.O. Box 257, Mozambique; 4 Provincial Health Research Unit, Xai-Xai P.O. Box 33, Mozambique; ismailposse@gmail.com (I.C.); amavic1@gmail.com (A.N.); 5 Provincial Health Directorate, Xai-Xai P.O. Box 33, Mozambique; srenaldojoao@gmail.com (S.R.J.); mulasimango@yahoo.com.br (M.S.); 6 Provincial Health Service, Xai-Xai P.O. Box 33, Mozambique; silviou2@gmail.com; 7 Faculty of Ethics and Human Sciences, São Tomás University of Mozambique, Xai-Xai P.O. Box 34, Mozambique; izaidino@yahoo.com; 8 District Health Service, Women and Social Action, Bilene P.O. Box 237, Mozambique; marcelinalindonde@gmail.com

**Keywords:** HIV, mental health, viral non-suppression, antiretroviral therapy, psychosocial indicators, Mozambique

## Abstract

**Highlights:**

**What are the main findings?**

- Endorsement of at least one psychosocial or mental health indicator was associated with higher crude odds of viral non-suppression, but the association was attenuated and statistically uncertain after adjustment for age, sex, and ART duration.
- Social isolation showed an exploratory association based on only seven participants, including two with viral non-suppression.

**What are the implications of the main findings?**

- The findings support further research using validated, culturally appropriate psychosocial and mental health measures.
- The study does not establish the clinical screening value of the composite or the benefit of psychosocial interventions for viral suppression.

**Abstract:**

**Background/Objectives**: Mental health challenges may compromise long-term HIV treatment outcomes, yet evidence linking psychosocial and mental health indicators with viral suppression remains limited in Mozambique. We examined the association between psychosocial and mental health indicators and viral non-suppression among adults receiving antiretroviral therapy (ART) in Gaza Province. **Methods**: We conducted a cross-sectional analytical study among 343 adults receiving ART at seven public health facilities in Xai-Xai District, Mozambique, in 2023–2024. The primary exposure was endorsement of at least one of three psychosocial and mental health indicators: social isolation, sadness, or use of medication for mental health problems. The primary outcome was viral non-suppression. Associations were estimated using Firth penalized logistic regression because of the small number of non-suppression events. **Results**: Overall, 41/343 participants (12.0%) reported at least one psychosocial or mental health indicator. Viral load results were available for 338 participants, of whom 19 (5.6%) had viral non-suppression. Viral non-suppression was more frequent among participants reporting at least one indicator than among those reporting none (12.2% vs. 4.7%). Reporting at least one indicator was associated with higher odds of viral non-suppression in unadjusted analysis (OR 2.95, 95% CI 1.04–8.34) and after adjustment for age and sex (aOR 2.97, 95% CI 1.05–8.38). After additional adjustment for ART duration, the association was attenuated and statistically uncertain (aOR 2.12, 95% CI 0.71–6.37). Social isolation showed the largest estimated crude odds ratio (OR 8.17, 95% CI 1.70–39.29), although estimates were imprecise. **Conclusions**: In the primary model adjusting for age, sex, and ART duration, the association was attenuated and imprecise (aOR 2.12, 95% CI 0.71–6.37). The findings are hypothesis-generating and should be interpreted in light of non-validated exposure measurement, substantial missing exposure information, and few non-suppression events.

## 1. Introduction

Human immunodeficiency virus (HIV) remains a major global public health challenge despite substantial progress in diagnosis, antiretroviral therapy (ART), and viral suppression. In 2025, an estimated 41.0 million people were living with HIV globally, 1.2 million people had newly acquired HIV, and approximately 570,000 people died from AIDS-related illnesses [1]. An estimated 32.1 million people were receiving ART, representing major gains in treatment access; nevertheless, only 74% of all people living with HIV were estimated to have achieved viral suppression [1]. Sustained viral suppression is essential for reducing HIV-related morbidity and mortality and preventing onward sexual transmission, making identification of factors that compromise long-term treatment success increasingly important [2,3].

The burden of HIV remains disproportionately concentrated in sub-Saharan Africa, particularly eastern and southern Africa [4]. Although the region has made substantial progress, the annual number of new HIV infections declined by 56% between 2010 and 2024 and AIDS-related deaths by 59%; eastern and southern Africa continues to carry a substantial share of the global HIV burden. In 2024, approximately 84% of people living with HIV in the region were receiving ART and 80% had achieved viral suppression, with important disparities by age and sex [4]. As ART programmes mature and increasing numbers of people live longer with HIV, maintaining viral suppression requires attention not only to access to medication but also to the psychosocial, behavioral, and structural factors that influence long-term engagement with treatment [3,5].

Mental health is increasingly recognized as an important component of successful long-term HIV care. People living with HIV experience a substantial burden of depression, anxiety, psychological distress, substance-use disorders, and other mental health conditions. A recent meta-analysis of studies among people living with HIV receiving ART in Africa estimated a pooled prevalence of depression of approximately 36%, although estimates varied considerably across settings and measurement approaches [6]. Earlier evidence from sub-Saharan Africa similarly demonstrated substantial heterogeneity, with estimates of depression among people living with HIV ranging from approximately 9% to 32% depending on the population and measurement instrument [7]. Mental health conditions may influence HIV outcomes through multiple pathways, including difficulties maintaining treatment routines, reduced motivation and self-care, social isolation, substance use, poorer engagement with healthcare services, and reduced capacity to mobilize social and material support [8,9].

The relationship between mental health and HIV treatment outcomes is particularly important in settings where mental health services remain insufficiently integrated into routine HIV care. Depression and other mental health conditions have been associated with poorer ART adherence and retention in care, potentially compromising sustained viral suppression [10]. A systematic review in sub-Saharan Africa found that people experiencing depressive symptoms had substantially lower odds of achieving good ART adherence than those without depressive symptoms [11]. Recognizing this relationship, the World Health Organization now recommends integration of care for depression, anxiety, and alcohol-use disorders within HIV service delivery, alongside interventions supporting long-term ART adherence [12]. However, mental health assessment and treatment remain insufficiently incorporated into routine HIV services in many low- and middle-income settings [11,13].

Mozambique provides an important context in which to examine this relationship. The country has one of the largest HIV epidemics globally. UNAIDS estimated that approximately 2.4 million people were living with HIV in Mozambique in 2023, including approximately 1.5 million adult women and 820,000 adult men. HIV prevalence among adults aged 15–49 years was estimated at 11.5%, while approximately 81,000 new HIV infections and 44,000 AIDS-related deaths occurred during the year [14]. Despite substantial expansion of ART services, achieving and sustaining viral suppression across this large population remains an important public health priority [5].

Evidence on mental health among people living with HIV in Mozambique is comparatively limited but suggests that the burden may be substantial [15]. A study conducted among 395 adults receiving ART in health facilities in Maputo and Nampula provinces found major depressive disorder in 27.3% of participants, suicidal ideation or behaviour in 15.4%, and alcohol-use disorder in 8.4%. Participants with any mental disorder had higher odds of reporting a missed ART dose than those without a mental disorder [16]. These findings highlight the relevance of mental health to HIV treatment in Mozambique, but important evidence gaps remain regarding the relationship between broader indicators of psychological and social vulnerability and objective treatment outcomes such as viral suppression, particularly outside Maputo and Nampula [16].

Understanding this relationship is especially relevant as HIV care increasingly shifts from ensuring ART initiation toward sustaining lifelong treatment and viral suppression [3]. Psychosocial and mental health indicators may not always present as a diagnosed psychiatric disorder; symptoms of sadness, social isolation, and other indicators of psychological distress may nevertheless identify individuals experiencing difficulties that could affect their long-term HIV care [17]. Examining these indicators within routine HIV services may therefore provide additional insight into potentially overlooked vulnerabilities among people receiving ART.

Mental health encompasses a wider domain than the presence or absence of a psychiatric diagnosis [10]. This study examines three available indicators relating to emotional experience, social connectedness, and mental health treatment [17]. Sadness is an emotional experience and does not, by itself, establish depression [6,7]. Social isolation concerns social connectedness, although the interpretation of this item depends on its questionnaire wording. Medication use for mental health problems describes treatment history and does not necessarily indicate current symptoms [10,12]. We therefore examine these items as distinct indicators and as an exploratory composite, without assuming that they measure a single mental health condition [17].

This study examined the association between psychosocial and mental health indicators and viral non-suppression among adults receiving ART in Xai-Xai District, Gaza Province, Mozambique. The primary exposure was an exploratory composite defined as endorsement of at least one of three indicators: sadness, social isolation, or use of medication for mental health problems. We also examined each indicator separately in exploratory analyses. We hypothesized that participants endorsing at least one indicator would have higher odds of viral non-suppression than those endorsing none.

## 2. Materials and Methods

### 2.1. Study Design and Setting

We conducted a cross-sectional analytical study among adults living with HIV receiving antiretroviral therapy (ART) in Xai-Xai District, Gaza Province, Mozambique. Participant enrolment and data collection began in October 2023 and continued for six months, during routine clinical follow-up visits, using a structured questionnaire administered by trained healthcare personnel. The present analysis examined the association between psychosocial and mental health indicators and viral non-suppression among adults receiving ART. The study was implemented across seven public health facilities providing HIV treatment services in Xai-Xai District: Centro de Saúde de Xai-Xai, Patrice Lumumba, Marian Ngoabi, Praia, Marian Ngoabi B, Chilaulene, and Zimilene.

### 2.2. Study Population and Participant Selection

The study population comprised adults aged ≥18 years living with HIV who registered in the HIV programme at participating health facilities between January and December 2021 and remained on ART at the time of enrolment. Registration at a participating facility was distinct from first ever ART initiation, which could have occurred before 2021. Participants were receiving routine follow-up through integrated HIV care clinics at participating public health facilities in Xai-Xai District. Potentially eligible participants were identified using the electronic ART database according to the predefined eligibility criteria. Eligible adults who were able to provide informed consent were invited to participate. Those who voluntarily agreed provided written informed consent before enrolment.

Patients receiving care at the Provincial Hospital of Xai-Xai, the prison health facility, and the Rapid Intervention Force (FIR) health center were excluded because these facilities either served populations with restricted liberty or provided services that differed from those evaluated in the study. Patients transferred into participating facilities from other health facilities during the study enrolment period were also excluded. Other exclusion criteria included follow-up through differentiated service delivery models, including sick child consultation (CCR), antenatal care (CPN), and adolescent-friendly services (SAAJ), with the exception of patients receiving services through the National Tuberculosis Control Programme (PNCTL). Patients receiving ART through treatment supporters or proxies were also excluded.

**Figure 1:**
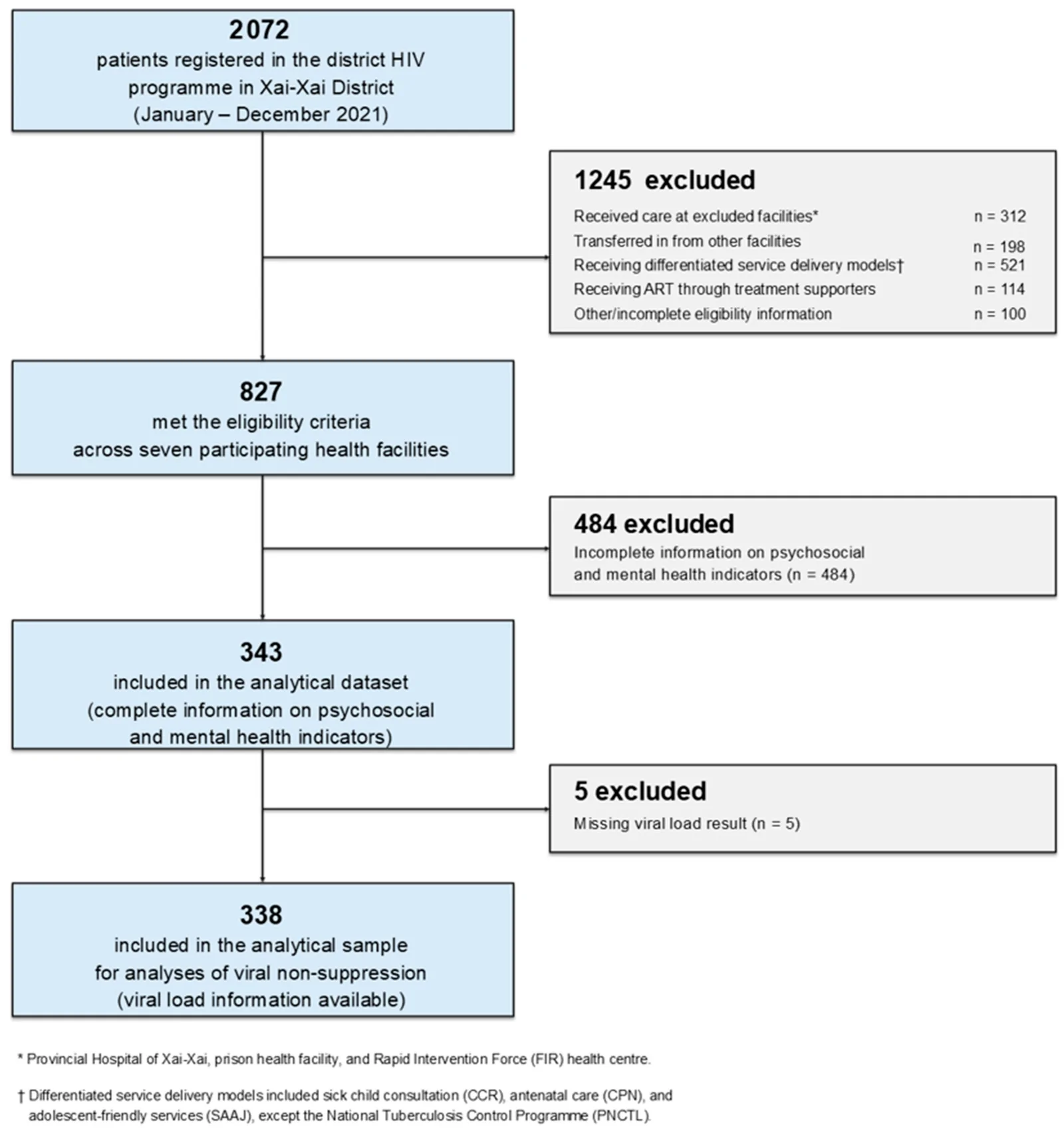
Flow diagram of participant selection for the analysis of psychosocial and mental health indicators and viral non-suppression among adults receiving antiretroviral therapy in Xai-Xai District, Mozambique, 2023–2024.

Between January and December 2021, 2072 patients registered in the district HIV programme, of whom 827 met the eligibility criteria across the seven participating health facilities. The analytical dataset included 343 participants with complete information on the three psychosocial mental health indicators used in the present analysis. Viral load information was available for 338 participants, who constituted the analytical sample for analyses of viral non-suppression (Figure 1).

Of the 827 otherwise eligible participants, 484 (58.5%) were excluded because complete information on the three psychosocial and mental health indicators was unavailable. Comparison data for these excluded participants were not available; therefore, differences between included and excluded participants could not be assessed. The reasons for incomplete exposure information could not be determined from the available records.

### 2.3. Data Collection

Data were collected through structured interviews conducted during routine clinical follow-up visits by trained healthcare personnel. The questionnaire captured sociodemographic, psychosocial, behavioral, clinical, and healthcare-related characteristics. Sociodemographic variables included age, sex, marital status, educational level, and place of residence. Psychosocial and behavioral characteristics included food security, family support, HIV status disclosure, disclosure to a partner, alcohol use, smoking, social isolation, sadness, and use of medication for mental health problems. Clinical information included duration on ART and viral load status. ART duration was calculated from the date of the first-ever ART initiation, including treatment received before registration at the participating facility. It therefore represented total time since ART initiation to date of study enrolment rather than time since facility registration or time on the current regimen.

### 2.4. Psychosocial and Mental Health Indicators

The primary exposure was an exploratory composite indicating endorsement of at least one of three questionnaire items concerning sadness, social isolation, or medication use for mental health problems. The composite was constructed for the present analysis by ATQ, IM and AN. The items were selected because they represented the available questionnaire information concerning emotional experience, social connectedness, and mental health treatment. These domains are related but distinct; their combination was not intended to establish a single underlying psychological construct. Each item was coded as 0 for no and 1 for yes. The primary exposure was defined as no endorsed indicators versus at least one endorsed indicator. Only participants with complete information on all three items were included. The composite has not undergone psychometric or diagnostic validation and should not be interpreted as a measure of mental disorder, symptom severity, or clinical screening status. Individual items were examined separately in exploratory analyses.

The questionnaire did not include a validated instrument assessing depression, anxiety, psychological distress, or social isolation. Ensuring the cultural and linguistic appropriateness of an existing instrument would have required translation, cultural adaptation, pilot testing, and validation, which were not feasible within the study timeframe. The present analysis was therefore restricted to the available individual questionnaire items. These items were not validated substitutes for established instruments, and interpretation is limited to the reported indicators and their exploratory composite.

The three psychosocial and mental health indicators were obtained from individual questions in the original structured questionnaire (Supplementary Table S1). Social isolation and sadness were assessed using questions referring to prolonged episodes of these experiences, although no specific recall period, frequency, or minimum duration was defined. Mental health medication use was assessed through a question about taking medication for mental illnesses, without specifying medication type, indication, or treatment duration. All three indicators were recorded as binary responses (yes/no). Medication use was self-reported in response to a yes/no question about taking medication for mental illnesses. No drug names, doses, indications, or prescribing diagnoses were collected, and no reference period was specified. This item therefore captures reported medication use without identifying the underlying condition or establishing current symptoms. The questionnaire items were not derived from a validated mental health instrument and were not designed to establish psychiatric diagnoses. Accordingly, the resulting composite represented endorsement of at least one of the three psychosocial and mental health indicators and was not interpreted as a validated measure of psychological distress or mental illness.

### 2.5. Outcome Definition

The primary outcome was viral non-suppression, defined as HIV RNA ≥ 1000 copies/mL; viral suppression was defined as HIV RNA < 1000 copies/mL, consistent with the threshold applied in Mozambique’s national HIV programme. Classification was based on the most recently documented single routine viral load measurement available in the study dataset. Repeat testing following enhanced adherence counselling was not required for this classification; therefore, the outcome represents viral non-suppression at a single measurement rather than confirmed virological failure. Viral load results were available for 338 of the 343 participants. The interval between viral load measurement and questionnaire administration was not available, limiting assessment of the temporal relationship between the reported indicators and viral load status.

An adherence variable was available in the original dataset, but its operational definition, assessment method, and derivation could not be established from the available documentation. Its classification was completely concordant with viral load status among the 338 participants with available measurements. We could not determine whether adherence had been assessed independently of viral load; therefore, this variable was excluded from the analyses and was not interpreted as an independent measure of medication-taking behaviour.

### 2.6. Covariates

Covariate selection was guided by the potential relationships of participant characteristics with both the exposure indicators and viral non-suppression. Age and sex were included as demographic covariates. ART duration, representing time since first ever ART initiation, was additionally included because treatment history may relate to current psychosocial experiences and virological outcomes. Education, marital status, and food insecurity were considered additional potential sources of confounding. However, the cross-sectional measurements did not establish whether some characteristics preceded the reported indicators or represented consequences or intermediates. With only 19 viral non-suppression events, adjustment was restricted to age, sex, and ART duration to limit model complexity. Residual confounding by other characteristics remains possible.

### 2.7. Statistical Analysis

Participant characteristics were summarized overall and according to whether participants reported none or at least one of the three psychosocial and mental health indicators. Continuous variables are presented as medians and interquartile ranges (IQRs), where appropriate, and categorical variables as frequencies and percentages. Age was compared between the two exposure groups using the Wilcoxon rank-sum (Mann–Whitney U) test. Given the small cell counts for several categorical variables, Fisher’s exact test was used to compare categorical characteristics between groups.

The prevalence of viral non-suppression was calculated for each exposure group and separately according to responses to each individual indicator. Because viral non-suppression was uncommon (19 events among 338 participants with available viral load results) and several exposure categories contained small numbers, associations were estimated using Firth-penalised logistic regression. This approach was selected to reduce small-sample estimation bias and problems arising from sparse data or separation in conventional maximum-likelihood logistic regression.

Univariable Firth logistic regression was used to estimate crude odds ratios (ORs) and 95% confidence intervals (CIs) for the association between reporting at least one psychosocial or mental health indicator and viral non-suppression, with participants reporting none as the reference group. Each individual indicator was also examined in a separate exploratory univariable model. Sequential multivariable models were then fitted. Model 1 adjusted for age and sex. Model 2 additionally adjusted for duration on ART and constituted the principal adjusted analysis. Crude and Model 1 estimates were reported to describe changes with adjustment. Given the small number of outcome events and sparse exposure categories, no additional covariate sensitivity analyses were conducted. Firth penalization was used to reduce small-sample estimation bias and separation problems, but it does not eliminate uncertainty arising from sparse data or unmeasured confounding. Results are reported as crude and adjusted odds ratios (aORs) with 95% CIs. All tests were two-sided, with *p* < 0.05 considered statistically significant. Analyses were conducted using Stata version 18.5; StataCorp, College Station, TX, USA.

### 2.8. Ethical Considerations

The study was conducted in accordance with applicable ethical principles for research involving human participants. All participants provided written informed consent before participation. The study received ethical approval from the Gaza institutional ethics committee (Reference No. 174/CIBS-Gaza/2023 on 9 October 2023). All data were anonymized prior to analysis.

## 3. Results

### 3.1. Participant Characteristics and Psychosocial and Mental Health Indicators

A total of 343 adults receiving ART were included in the analysis. The median age was 35 years (IQR 29–43), and 170 (49.6%) participants were female. At least one of the three indicators was reported by 41/343 participants (12.0%); 302/343 (88.0%) reported none. Among participants with psychosocial and mental health indicators, 39 reported one indicator and two reported two indicators. No participant reported all three indicators.

Participant characteristics according to psychosocial and mental health indicator status are presented in Table 1. Age did not differ significantly between participants with and without psychosocial and mental health indicators (median 35 [IQR 29–47] vs. 34 [IQR 29–42] years; *p* = 0.658), and the distribution by sex was also similar (*p* = 0.869). Marital status differed significantly between groups (*p* = 0.001). In particular, 9.8% of participants with psychosocial and mental health indicators were separated or divorced compared with 1.0% of those endorsing none, whereas widowhood was less frequent among participants endorsing at least one indicator (2.4% vs. 16.9%).

Educational attainment also differed according to psychosocial and mental health indicator status (*p* < 0.001). Higher education was reported by 63.4% of participants endorsing at least one indicator compared with 26.8% of those endorsing none. Place of residence was not significantly associated with psychosocial and mental health indicators (*p* = 0.217).

Food insecurity was more frequent among participants endorsing at least one indicator: 78.1% were food insecure compared with 48.7% of participants endorsing none (*p* < 0.001). In contrast, family support, HIV status disclosure, and disclosure to a partner did not differ significantly between groups (*p* = 1.000, *p* = 0.603, and *p* = 0.218, respectively). Alcohol use was also similar between groups (43.9% vs. 48.7%; *p* = 0.619). None of the participants endorsing at least one indicator reported smoking, compared with 11.3% of those endorsing none (*p* = 0.022).

Duration since first-ever ART initiation differed markedly between the groups (*p* < 0.001). Among participants endorsing at least one psychosocial or mental health indicator, 37/41 (90.2%) had received ART for more than five years, compared with 132/302 (43.7%) among those endorsing none. This imbalance is relevant to interpretation of the adjusted association with viral non-suppression.

### 3.2. Psychosocial and Mental Health Indicators and Viral Non-Suppression

Viral load results were available for 338 of the 343 participants. Overall, 19/338 (5.6%) participants were virally non-suppressed. Viral non-suppression was more frequent among participants endorsing at least one indicator than among those endorsing none (5/41 [12.2%] vs. 14/297 [4.7%]). In univariable Firth penalized logistic regression, participants endorsing at least one indicator had nearly three times the odds of viral non-suppression compared with those endorsing none (OR 2.95, 95% CI 1.04–8.34; *p* = 0.042).

In an exploratory univariable analysis, social isolation had an OR of 8.17 for viral non-suppression (95% CI 1.70–39.29). Only seven participants reported isolation, of whom two were non-suppressed. This sparse, unadjusted estimate requires confirmation. Two of the seven participants reporting social isolation (28.6%) were virally non-suppressed, compared with 17/331 (5.1%) of those not reporting isolation. In Firth regression, social isolation was associated with higher odds of viral non-suppression (OR 8.17, 95% CI 1.70–39.29; *p* = 0.009), although the estimate was imprecise, as reflected by the wide confidence interval and small number of participants reporting isolation.

Viral non-suppression occurred in 3/27 (11.1%) participants reporting sadness compared with 16/311 (5.1%) of those not reporting sadness. The corresponding odds ratio was elevated but not statistically significant (OR 2.56, 95% CI 0.75–8.71; *p* = 0.133). Similarly, one of nine participants (11.1%) reporting use of mental health medication was virally non-suppressed compared with 18/329 (5.5%) of those not reporting mental health medication use; this association was also not statistically significant (OR 2.97, 95% CI 0.49–17.94; *p* = 0.235) (Table 2).

### 3.3. Multivariable Association Between Psychosocial and Mental Health Indicators and Viral Non-Suppression

Table 3 presents the sequential Firth penalized logistic regression models examining the association between psychosocial and mental health indicators and viral non-suppression. In the principal model adjusting for age, sex, and ART duration, endorsement of at least one psychosocial or mental health indicator had an adjusted odds ratio of 2.12 for viral non-suppression (95% CI 0.71–6.37; *p* = 0.178). The confidence interval included the null and indicated substantial uncertainty. The corresponding crude and age- and sex-adjusted estimates were 2.95 (95% CI 1.04–8.34) and 2.97 (95% CI 1.05–8.38), respectively (Table 3).

After additional adjustment for duration on ART, the magnitude of the association between psychosocial and mental health indicators and viral non-suppression attenuated from an aOR of 2.97 to 2.12 and was no longer statistically significant (aOR 2.12, 95% CI 0.71–6.37; *p* = 0.178). In the fully adjusted model, age (aOR 0.99 per year, 95% CI 0.94–1.03; *p* = 0.625) and sex (female vs. male: aOR 0.95, 95% CI 0.38–2.37; *p* = 0.916) remained unassociated with viral non-suppression. Compared with participants who had received ART for less than one year, those receiving ART for 1–5 years had lower estimated odds of viral non-suppression (aOR 0.14, 95% CI 0.02–1.06; *p* = 0.057), although the confidence interval included the null. The corresponding estimate for participants receiving ART for more than five years was aOR 0.39 (95% CI 0.06–2.52; *p* = 0.324). Overall, endorsement of at least one indicator was associated with higher odds of viral non-suppression in crude and age- and sex-adjusted analyses. After additional adjustment for ART duration, the estimate was attenuated and statistically uncertain, with wide confidence intervals.

## 4. Discussion

In this cross-sectional study of adults receiving ART in Gaza Province, Mozambique, approximately one in eight participants endorsed at least one of three psychosocial and mental health indicators: social isolation, sadness, or use of medication for mental health problems. Viral non-suppression occurred in 12.2% of participants endorsing at least one indicator and 4.7% of those endorsing none. Endorsement was associated with approximately three times the odds of viral non-suppression in unadjusted analysis and after adjustment for age and sex. However, the principal estimate was attenuated and statistically uncertain after additional adjustment for ART duration. Social isolation had the largest estimated crude odds ratio among the individual indicators, but this exploratory finding was based on only seven participants, including two with viral non-suppression. These findings warrant further investigation using validated measures and longitudinal designs.

Overall, 12.0% of participants endorsed at least one of the three psychosocial and mental health indicators, and only two endorsed more than one. This proportion should not be interpreted as the prevalence of mental disorders. The items were not derived from a validated screening instrument for depression, anxiety, or other psychiatric disorders and describe distinct experiences and treatment histories. The reported items describe different experiences and treatment histories. Their endorsement may provide a reason for further enquiry during care, but this study did not establish their diagnostic accuracy, predictive performance, or usefulness for directing clinical decisions. Medication use, in particular, may reflect previous treatment and access to services rather than current symptoms [10,12].

The principal finding was the higher occurrence of viral non-suppression among participants endorsing at least one indicator. The approximately three-fold higher odds observed in the crude analysis persisted after adjustment for age and sex, suggesting that the association was not explained by these basic demographic characteristics. Mental health difficulties may affect HIV treatment outcomes through several interconnected pathways, including reduced motivation for self-care, difficulties maintaining daily treatment routines, impaired engagement with healthcare services, and reduced capacity to mobilize social and material resources [3,18,19]. Conversely, difficulties with HIV treatment or concerns about treatment response may themselves contribute to emotional distress [20]. Because psychosocial and mental health indicators and viral load were assessed within a cross-sectional study, the temporal direction of these relationships cannot be established. The findings therefore support an association between psychosocial and mental health indicators and viral non-suppression rather than a causal effect of psychosocial and mental health indicators on virological outcomes.

ART duration was an important consideration in interpreting the association. Participants endorsing at least one indicator were disproportionately represented among those receiving ART for more than five years. Adding ART duration to the age- and sex-adjusted model attenuated the estimated association from an aOR of 2.97 (95% CI 1.05–8.38) to 2.12 (95% CI 0.71–6.37). The principal adjusted estimate was therefore statistically uncertain. This attenuation is compatible with confounding by treatment history, but does not establish its causal role. Sparse data and the wide confidence interval further limit interpretation. Longer engagement with HIV care may create cumulative psychosocial challenges that are not captured by conventional measures of adherence or clinical status. Long-term ART care therefore provides an important context within which mental health and virological outcomes should be considered [21,22,23].

Food insecurity was another notable characteristic of participants endorsing at least one indicator. More than three-quarters of participants endorsing at least one indicator were food insecure, compared with approximately half of those without. Material hardship, uncertainty about access to food, and the challenges of taking lifelong treatment in conditions of socioeconomic insecurity may contribute to psychological distress [24,25]. At the same time, poor mental wellbeing can affect an individual’s ability to work, maintain social relationships, and access resources [26]. These findings support viewing mental health within HIV care as part of a broader psychosocial and socioeconomic context rather than as an isolated clinical condition.

Differences in marital status and education were also observed between the exposure groups. Participants who were separated or divorced were disproportionately represented among those endorsing at least one indicator, although the absolute number was small. Previous research among people living with HIV has identified interpersonal factors, including reduced social support, relationship disruption, and social isolation, as important correlates of psychological distress and poor mental health outcomes [20,27,28]. However, the cross-sectional nature of the present study and the small subgroup sizes preclude further inference regarding the direction or nature of these relationships. The higher proportion of participants with higher educational attainment among those with psychosocial and mental health indicators was less expected and highlights the complexity of mental health risk among people living with HIV [29]. Psychosocial and mental health indicators may occur across diverse socioeconomic groups and should not be assumed to be restricted to conventionally defined socially disadvantaged populations.

Social isolation emerged as the strongest individual mental health signal. Viral non-suppression occurred in 28.6% of participants reporting isolation compared with 5.1% among those who did not report isolation, and the corresponding Firth regression estimate suggested substantially higher odds of non-suppression. However, only seven participants reported social isolation and only two of these were virally non-suppressed. The resulting confidence interval was consequently very wide. This finding should therefore be regarded as exploratory rather than evidence of a precise independent association. Nevertheless, the magnitude of the observed difference warrants further investigation. Social connectedness may be particularly relevant to HIV care because treatment is managed largely outside healthcare facilities and may depend on practical assistance, emotional support, disclosure relationships, and sustained engagement with care [30,31]. Future studies should investigate social isolation using validated measures and larger samples to determine whether it represents an important marker of vulnerability to poor HIV outcomes.

Routine HIV services may provide opportunities to assess psychosocial concerns and mental health needs [12]. However, this study did not evaluate a screening strategy, referral pathway, or intervention, and it does not establish that these approaches would improve viral suppression. The exploratory composite should not be adopted as a clinical screening tool on the basis of these findings. Further research should evaluate validated, culturally appropriate assessment tools and feasible care pathways in Mozambique, including their acceptability, resource requirements, and effects on patient outcomes.

This study also points to several priorities for future research. Prospective studies should use validated and culturally appropriate measures of depression, anxiety, psychological distress, and social isolation and examine their relationship with subsequent viral load outcomes. Future research should address the measurement limitations of this analysis by using instruments with demonstrated validity and cultural appropriateness in the intended population. The present composite should not be adopted as a clinical screening tool on the basis of these findings. Longitudinal designs would help establish whether the reported experiences precede viral non-suppression, develop following treatment difficulties, or have bidirectional relationships with treatment outcomes. Larger studies would also permit investigation of interactions between mental health and food insecurity, social support, gender, treatment duration, and other structural vulnerabilities. Qualitative research could complement these analyses by exploring how adults living with HIV in Mozambique understand emotional distress, loneliness, treatment fatigue, social support, and their influence on long-term engagement with HIV care.

The study has several limitations. Its cross-sectional design prevents determination of temporality or causality between psychosocial and mental health indicators and viral non-suppression. The exploratory composite was constructed from three available questionnaire items rather than a validated mental health screening instrument and should not be interpreted as a measure of a specific psychiatric disorder. Some individual indicators were uncommon, particularly social isolation and mental health medication use, resulting in sparse cells and wide confidence intervals despite the use of Firth penalized logistic regression. A substantial proportion of otherwise eligible participants (484/827; 58.5%) were excluded because of incomplete exposure information. Comparison data for excluded participants were unavailable, preventing assessment of whether the analytical sample differed systematically from the eligible population. Selection related to both the exposure indicators and viral load status could have biased the estimated association in either direction. The magnitude and direction of this potential bias remain unknown. Consequently, the reported indicator frequencies and associations apply to the analytical sample and may not represent all eligible patients or the wider population receiving ART.

The small number of non-suppression events constrained covariate adjustment. Although the principal model included age, sex, and ART duration, residual confounding by socioeconomic circumstances, education, marital status, and other factors remains possible. No additional sensitivity analyses were conducted, so robustness to alternative covariate specifications was not assessed. The attenuation after adjustment for ART duration does not, by itself, establish the underlying causal relationships. Mental health and behavioral characteristics were self-reported and may have been affected by social desirability or reporting bias. Viral load information was unavailable for five participants, although this represented a small proportion of the study population. An independently documented measure of ART adherence was unavailable. We therefore could not examine adherence as a potential pathway linking the psychosocial and mental health indicators with viral non-suppression. Finally, participants were recruited from public HIV treatment facilities in one district of Gaza Province, which may limit generalizability to other populations and regions of Mozambique.

## 5. Conclusions

Among adults receiving ART in Gaza Province, participants endorsing at least one of three psychosocial and mental health indicators had a higher observed prevalence of viral non-suppression. After adjustment for age, sex, and ART duration, the association was attenuated and imprecise (aOR 2.12, 95% CI 0.71–6.37), providing insufficient evidence of an independent association. Non-validated exposure measurement, substantial exclusion due to missing exposure information, and few outcome events limit interpretation. These findings are hypothesis-generating and support prospective research using validated, culturally appropriate measures.

## Figures and Tables

**Table 1:** Sociodemographic, psychosocial, behavioral and clinical characteristics according to psychosocial and mental health indicators among adults receiving ART in Gaza Province, Mozambique (*N* = 343).

Characteristic	Overall *N* = 343, *n* (%)	No Endorsed Indicators, *n* = 302 *n* (%)	At Least One Endorsed Indicator, *n* = 41 *n* (%)	*p*-Value
Age, years, median (IQR)	35 (29–43)	34 (29–42)	35 (29–47)	0.658
Sex				0.869
Male	173 (50.4)	153 (50.7)	20 (48.8)	
Female	170 (49.6)	149 (49.3)	21 (51.2)	
Marital status				0.001
Single	127 (37.0)	110 (36.4)	17 (41.5)	
Married/cohabiting	157 (45.8)	138 (45.7)	19 (46.3)	
Separated/divorced	7 (2.0)	3 (1.0)	4 (9.8)	
Widowed	52 (15.2)	51 (16.9)	1 (2.4)	
Education				<0.001
Primary	41 (12.0)	41 (13.6)	0 (0.0)	
Secondary	181 (52.8)	166 (55.0)	15 (36.6)	
Higher	107 (31.2)	81 (26.8)	26 (63.4)	
Other	14 (4.1)	14 (4.6)	0 (0.0)	
Residence				0.217
Urban	317 (92.4)	281 (93.1)	36 (87.8)	
Rural	26 (7.6)	21 (7.0)	5 (12.2)	
Food security				<0.001
Food secure	164 (47.8)	155 (51.3)	9 (22.0)	
Food insecure	179 (52.2)	147 (48.7)	32 (78.1)	
Family support				1.000
No	42 (12.2)	37 (12.3)	5 (12.2)	
Yes	301 (87.8)	265 (87.8)	36 (87.8)	
HIV status disclosure				0.603
No	8 (2.3)	8 (2.6)	0 (0.0)	
Yes	335 (97.7)	294 (97.4)	41 (100.0)	
Disclosure to partner				0.218
No	68 (19.8)	63 (20.9)	5 (12.2)	
Yes	275 (80.2)	239 (79.1)	36 (87.8)	
Alcohol use				0.619
No	178 (51.9)	155 (51.3)	23 (56.1)	
Yes	165 (48.1)	147 (48.7)	18 (43.9)	
Smoking				0.022
No	309 (90.1)	268 (88.7)	41 (100.0)	
Yes	34 (9.9)	34 (11.3)	0 (0.0)	
Duration since first-ever ART initiation				<0.001
<1 year	8 (2.3)	8 (2.6)	0 (0.0)	
1–5 years	166 (48.4)	162 (53.6)	4 (9.8)	
>5 years	169 (49.3)	132 (43.7)	37 (90.2)	

Values are *n* (%) unless otherwise indicated. The exposure groups were defined by endorsement of none versus at least one of three psychosocial and mental health indicators: social isolation, sadness, or use of medication for mental health problems. *p*-values were calculated using Fisher’s exact test for categorical variables and the Mann–Whitney U test for age.

**Table 2:** Association of the exploratory composite and individual psychosocial and mental health indicators with viral non-suppression among adults receiving ART in Mozambique.

Exposure	Viral Suppression, *n*/*N* (%)	Viral Non-Suppression, *n*/*N* (%)	Crude OR (95% CI) *	*p*-Value ^†^
Psychosocial and mental health indicators				
No endorsed indicators	283/297 (95.3)	14/297 (4.7)	1.00	
At least one endorsed indicator	36/41 (87.8)	5/41 (12.2)	2.95 (1.04–8.34)	0.042
Social isolation				
No	314/331 (94.9)	17/331 (5.1)	1.00	
Yes	5/7 (71.4)	2/7 (28.6)	8.17 (1.70–39.29)	0.009
Sadness				
No	295/311 (94.9)	16/311 (5.1)	1.00	
Yes	24/27 (88.9)	3/27 (11.1)	2.56 (0.75–8.71)	0.133
Mental health medication use				
No	311/329 (94.5)	18/329 (5.5)	1.00	
Yes	8/9 (88.9)	1/9 (11.1)	2.97 (0.49–17.94)	0.235

* Odds ratios and 95% CIs estimated using separate univariable Firth penalized logistic regression models. ^†^ *p*-values correspond to the Firth regression coefficients rather than Fisher’s exact tests. Viral-load data were available for 338 of 343 participants.

**Table 3:** Association between psychosocial and mental health indicators and viral non-suppression.

Variable	Crude OR (95% CI)	*p*-Value	Model 1 aOR (95% CI)	*p*-Value	Model 2 aOR (95% CI)	*p*-Value
Psychosocial and mental health indicators						
No endorsed indicators	1.00	-	1.00	-	1.00	-
At least one endorsed indicator	2.95 (1.04–8.34)	0.042	2.97 (1.05–8.38)	0.040	2.12 (0.71–6.37)	0.178
Age, per year	-	-	0.98 (0.94–1.03)	0.509	0.99 (0.94–1.03)	0.625
Sex						
Male	-	-	1.00	-	1.00	-
Female	-	-	0.89 (0.36–2.21)	0.804	0.95 (0.38–2.37)	0.916
Duration on ART						
<1 year	-	-	-	-	1.00	-
1–5 years	-	-	-	-	0.14 (0.02–1.06)	0.057
>5 years	-	-	-	-	0.39 (0.06–2.52)	0.324

Model 1: adjusted for age and sex. Model 2: adjusted for age, sex, and duration on ART is the principal analysis. OR = odds ratio; aOR = adjusted odds ratio; CI = confidence interval. Estimates were obtained using Firth penalized logistic regression.

## Data Availability

The datasets generated and/or analyzed during the current study are not publicly available because they contain potentially identifiable participant information and are subject to ethical and institutional restrictions. De-identified data may be made available from the corresponding author upon reasonable request.

## Supplementary Materials

The following supporting information can be downloaded at https://www.mdpi.com/article/10.3390/healthcare14213679/s1, Table S1: Psychosocial and mental health items and composite definition.

## Abbreviations

The following abbreviations are used in this manuscript:

ART	antiretroviral therapy
CI	confidence interval
CCR	sick child consultation
CPN	antenatal care
FIR	Rapid Intervention Force
HIV	human immunodeficiency virus
IQR	interquartile range
OR	odds ratio
aOR	adjusted odds ratio
PLHIV	people living with HIV
PNCTL	National Tuberculosis Control Programme
SAAJ	adolescent-friendly health services
VL	viral load
WHO	World Health Organization
