# Supplementary material for: Psychosocial and Mental Health Indicators and Viral Non-Suppression Among Adults Receiving Antiretroviral Therapy in Gaza Province, Mozambique: A Cross-Sectional Study
Source: Healthcare (Basel). 2026 Oct 30;14(21):3679. doi: 10.3390/healthcare14213679 (PMC13762699; doi:10.3390/healthcare14213679)
Supplement: Supplementary file 1 [file healthcare-14-03679-s001.zip › healthcare-4616533-supplementary.pdf]

Table S1 Psychosocial and mental health items and composite definition

Original Portuguese wording, English translations, coding, and measurement scope of the three items used in the exploratory composite.

Item and source	Original wording and English translation	Timing and operational interpretation
Isolation<br>Question 20<br><br>Yes 7; no 336	Portuguese: "Apresenta episódios prolongados de sintomas como isolamento?"<br><br>English: "Do you experience prolonged episodes of symptoms such as isolation?"	No fixed recall period or duration threshold is specified. "Prolonged" is not quantified. A positive response indicates reported isolation; the item does not distinguish objective social isolation, perceived isolation, loneliness, or social withdrawal.
Sadness<br>Question 21<br><br>Yes 27; no 316	Portuguese: "Apresenta episódios prolongados de sintomas como tristeza?"<br><br>English: "Do you experience prolonged episodes of symptoms such as sadness?"	No fixed recall period or duration threshold is specified. "Prolonged" is not quantified. A positive response indicates reported sadness; it does not establish depression, symptom severity, or a psychiatric diagnosis.
Medication use for mental illness<br>Question 24<br><br>Yes 9; no 334	Portuguese: "Consome Medicamentos para doenças mentais?"<br><br>English: "Do you take medication for mental illnesses?"	Present-tense wording, without a defined reference period. A positive response indicates reported medication use. No information on drug names, doses, indications, or prescribing diagnoses was collected. Medication use cannot be attributed to a specific mental health condition.

Composite definition: Using the binary data-sheet values, the three items are summed (possible range 0-3). No endorsed indicators is defined as a sum of 0; at least one endorsed indicator as a sum ≥1. All 343 populated participant records have values for all three items: 302 have a sum of 0, 39 a sum of 1, and two a sum of 2. None has a sum of 3. Missing responses must not be recoded as no.

Measurement limitations: The items and composite are not validated diagnostic or screening measures. Portuguese wording is reproduced from the workbook codebook; English translations are supplied for reporting and are not validated translations.
